# Sirolimus increases tissue factor expression but not activity in cultured human vascular smooth muscle cells

**DOI:** 10.1186/1471-2261-5-22

**Published:** 2005-07-15

**Authors:** Shengsi Zhu, Hema Viswambharan, Thusitha Gajanayake, Xiu-Fen Ming, Zhihong Yang

**Affiliations:** 1 Vascular Biology, Department of Medicine, Division of Physiology,University of Fribourg, Rue du Musée 5, CH-1700 Fribourg, Switzerland; 2 Cardiovascular Department, 1^st ^Affiliated Hospital of Dalian Medical University, Dalian, P.R. China

## Abstract

**Background:**

Sirolimus-eluting stents (CYPHER stents) demonstrated remarkable efficacy in reducing restenosis rates in patients with coronary artery disease. There is a concern of sub-acute and late stent thrombosis. Tissue factor (TF) is critical in thrombosis. This study investigated the effect of sirolimus on TF expression and activity in cultured human vascular smooth muscle cells (SMCs).

**Methods:**

SMCs were cultured from human saphenous veins and aortas. Quiescent cells were stimulated with sirolimus (0.1 – 20 ng/ml) over 24 hours. Cellular TF expression and activity released into culture medium were measured. The effect of sirolimus on activation of *m*ammalian *t*arget *o*f *r*apamycin (mTOR) was measured by phosphorylation of the substrate p70s6k at T389, and activation of RhoA was measured by pull-down assay.

**Results:**

Sirolimus increased TF protein level in cultured human SMCs in a concentration and time-dependent manner (about 2-fold, p < 0.01) reaching maximal effect at 5 ng/ml. The stimulation of TF expression by sirolimus was associated with inhibition of basal activity of mTOR. No effects of sirolimus on RhoA or p38mapk activation that are positive regulators of TF in vascular wall cells were observed. The stimulation of TF expression by sirolimus (20 ng/ml) was prevented by the HMG-CoA reductase inhibitor fluvastatin (1 μmol/L). However, no increase in TF activity released from SMC into culture medium was observed after sirolimus treatment.

**Conclusion:**

Although sirolimus stimulates TF protein expression in human SMC associated with inhibition of mTOR, it does not enhance TF activity released from the cells, suggesting a relatively safe profile of CYPHER stents. The inhibition of TF expression by fluvastatin favors clinical use of statins in patients undergoing coronary stenting.

## Background

Since the first human study with sirolimus (rapamycin)-eluting stents (Cordis CYPHER™ stent) by Sousa [1], considerable promise of sirolimus-eluting stents for reducing restenosis rates and clinical parameters was subsequently demonstrated by several randomized clinical trials [2-7]. The mechanism of inhibition of restenosis by sirolimus has been suggested to be attributed to the blockade of smooth muscle cell (SMC) cycle progression from G1 to S phase via inhibition of the protein kinase, *m*ammalian *t*arget *o*f *r*apamycin (mTOR)[8].

Despite the promising results on restenosis rates, there is concern that drug-eluting stents may be associated with increased thrombosis rates. Although stent thrombosis associated with sirolimus-eluting stents has been reported in several clinical trials, it remains a rare event and is not higher in patients receiving bare metal stents [2-4,9,10]. Pooled analysis of clinical trials does not reveal a higher incidence of stent thrombosis, suggesting a relative safe profile of drug-eluting stents at least under the condition of anti-platelet regiment [11]. However, individual case reports generated some suspicion that drug-eluting stents may be prone to thrombosis [12]. In a report, four cases of late coronary thrombosis related to drug-eluting stents were presented, all of them occurred shortly after anti-platelet therapy was interrupted [12], and in two patients who received both a bare metal stent and a sirolimus-eluting stent, only the sirolimus-eluting stents were closed due to thrombosis, while the bare metal stents remained open in the same patients [12]. Based on the controversial reports and concerns, we analyzed whether sirolimus per se exerts some adverse effects related to thrombosis in vascular cells namely smooth muscle cells.

Tissue factor (TF) plays an important role in thrombosis and acute coronary syndromes [13]. It is the principle initiator of extrinsic coagulation pathway activating thrombin and generating fibrin leading to thrombus formation. Recent study suggests that aberrant TF expression in the vascular wall cells plays a crucial role in triggering intravascular thrombosis [14]. Under non-stimulated conditions, vascular wall cells i.e. endothelial cells and SMCs express negligible or low level of TF that can be up-regulated by cytokines and thrombin [15-17]. Several intracellular signal transduction mechanisms have been demonstrated to be involved in the regulation of TF expression. The small G-protein RhoA and the protein kinase p38mapk are positive regulators, whereas phosphatidylinositol 3-kinase (PI3-K) negatively regulates TF expression in vascular wall cells [17].

The HMG CoA reductase inhibitors or statins reduce cardiovascular events in patients with coronary heart disease [18]. The non-cholesterol lowering effects i.e. pleiotropic effects of statins seem to play important roles [19]. Experimental studies demonstrate that statins increase eNOS expression in endothelial cells, inhibit TF expression in SMC via inhibition of Rho/ROCK pathway [16,20]. Hence, the present study is aimed to investigate whether sirolimus could promote TF expression in human SMC, and whether this is associated with an increased TF activity. The effects of statin such as fluvastatin on TF expression in SMC were also investigated.

## Methods

### Materials

Sirolimus was purchased from Calbiochem (Lucerne, Switzerland); fluvastatin was kindly provided by Novartis (Basel, Switzerland); tumor necrosis factor-α (TNF-α) was purchased from R & D, France); monoclonal mouse anti-TF antibody and tissue factor activity kit were purchased from American Diagnostica Inc (Socochim, Lausanne, Switzerland); anti-tubulin and all the other chemicals for immunoblotting were purchased from Sigma (Buchs, Switzerland); anti-phospho p70s6k (T389) was from Cell Signaling Technology. Alkaline phosphatase (AP)-conjugated anti-mouse IgG and BCIP/NBT substrate for AP were from Interchim (Chemie Brunschwig AG, Basel, Switzerland).

### SMC and endothelial cell culture

SMC were isolated and cultured from human saphenous veins [20] and human aortic SMC were kindly provided by Dr. Therese Resink (University of Basel, Switzerland). Endothelial cells from human umbilical veins were isolated as previously described [17].

### TF expression

Cells were rendered quiescent for 24 hours in DMEM containing 0.2% BSA before they were treated with sirolimus (20 ng/ml, 24 hours), a concentration which fully inhibits mTOR/p70s6k pathway as previously shown [21]. To study the effect of fluvastatin on sirolimus-induced TF expression, the cells were pre-incubated with fluvastatin (1 μmol/L) for 60 minutes. Cell lysates were prepared as described [17]. 30 μg extracts were used for immunoblotting of TF expression [17]. Tubulin expression was used to ensure equal protein loading. Quantification was performed using NIH Image-J software. TF expression was expressed as percentage changes of the basal level.

### TF activity in cell conditioned medium

2 × 10^-5 ^cells/ml were seeded onto each dish for overnight attachment. Cells were then rendered quiescent in phenol-red free DMEM medium containing 0.2% BSA for 24 hours and then treated with sirolimus (20 ng/ml; 24 hours) as described above except that conditioned medium was collected and TF activity was measured as instructed by the manufacturer. Briefly, the same amount of conditioned medium (25 μl) was incubated in the presence of Factor VIIa and Factor X for 15 minutes in a 96-well plate, after which the substrate was added and further incubated for another 60 minutes before the reaction was stopped with glacial acetic acid and color reaction was measured with a microplate reader at 405 nm. TF activity is expressed in picomolar obtained from the standard curve.

### RhoA activation

The activation of RhoA was assessed by pull-down assay in the cells stimulated with sirolimus (20 ng/ml) over one hour as described [17]. Briefly, SMCs were washed with ice-cold Tris-buffered saline and lysed in RIPA buffer (50 mmol/L Tris, pH 7.2, 1% Triton X-100, 0.5% sodium deoxycholate, 0.1% SDS, 500 mmol/L NaCl, 10 mmol/L MgCl_2_, 10 μg/ml each of leupeptin and aprotinin, and 1 mmol/L PMSF). 200 μg of cell lysates were incubated with 10 μg of GST-TRBD beads at 4°C for 60 min. The beads were washed four times with buffer B (Tris-buffer containing 1% Triton X-100, 150 mmol/L NaCl, 10 mmol/L MgCl_2_, 10 μg/ml each of leupeptin and aprotinin, and 0.1 mmol/L PMSF). Bound RhoA proteins were then detected by immunoblotting using a monoclonal antibody against RhoA (Santa Cruz Biotechnology). The total amount of RhoA in cell lysates was used as a control for the cross-comparison of RhoA activity (level of GTP-bound RhoA).

### mTOR activation

mTOR activation was examined by immunoblotting measuring p70s6k phosphorylation at T389 in quiescent cells with or without sirolimus treatment (20 ng/ml, 1 hour). 40 μg cell extracts were subjected to 8% SDS-PAGE and phosphorylated p70s6k was detected using anti-phospho-p70s6k (T389) antibody. Activation of p70s6k was calculated as ratio of phospho-p70s6k against tubulin.

### Statistic analysis

All data were expressed as mean ± SEM and one way analysis of variance (ANOVA) with Bonferroni's post-test was used for statistical analysis. A two-tailed value of *p *≤ 0.05 was considered statistically significant.

## Results and discussion

**Figure 1:**
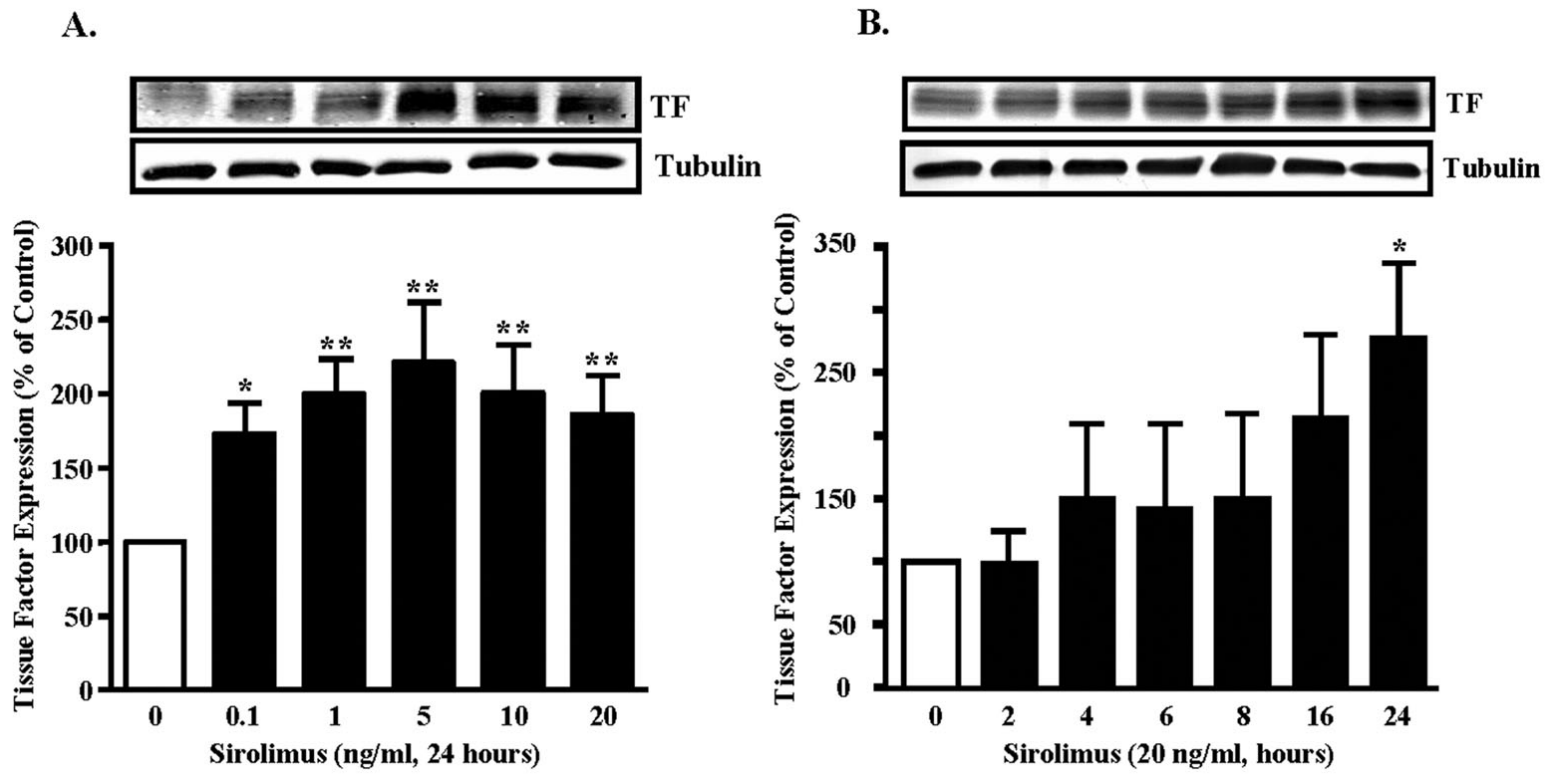
**Sirolimus up-regulates tissue factor (TF) expression in SMC. **(A). Sirolimus enhances TF expression in a concentration- and (B) time-dependent manner in human SMC. n = 6, * = p < 0.05 vs. control, ** = p < 0.01 vs. control.

Sirolimus-eluting stents have demonstrated remarkable clinical efficacy in reducing restenosis rates in the short-to-medium term [1-6]. There is a concern of subacute and late stent thrombosis [22-24] although individual clinical trials and pooled analysis of all the randomized trials showed no evidence of increase in stent thrombosis with drug-eluting stents as compared to the bare-metal stents in the short-to-medium term [2-4,9,10,25]. Some clinical experiences suggest that sirolimus-eluting stents may be prone to thrombosis [12,22,23,26,27]. In an earlier report [22], a case of late stent thrombosis associated with sirolimus-eluting stent was noticed 2 weeks after the patient stopped anti-platelet therapy. More recently, a clinical report presented four cases of late coronary thrombosis related to drug-eluting stents that occurred several months after coronary intervention [12]. It raises much concern by the observation that in two patients who received both a bare metal stent and a sirolimus-eluting stent, only the drug-eluting stents were closed due to late stent thrombosis which developed shortly after anti-platelet therapy was interrupted, whereas the bare metal stents in the same patients remained open [12]. This observation may indicate that sirolimus could exert pro-thrombotic effects, in particular, when anti-platelet therapy was discontinued. Adverse effects of sirolimus related to thrombosis have been documented *in vitro *experiments and also *in vivo *in an animal model [28-30]. Sirolimus has been reported to inhibit endothelium-dependent relaxations in porcine coronary arteries [28]. Inhibition of endothelialization by sirolimus has also been shown in human necropsy specimens and in animal models [31,32]. Moreover, stimulation or facilitation of platelet aggregation and secretion by sirolimus has also been demonstrated [29]. The pro-thrombotic effect of sirolimus was also demonstrated in a rat model of synthetic vascular grafts [30]. In our present study, we demonstrated that in human SMC, sirolimus increased TF protein level at a low concentration i.e. 0.1 ng/ml, which reached the maximal effect at 5 ng/ml (Fig. 1A). This concentration is in the range of clinical settings, since systemic level of sirolimus was reported to be in the range of 1~2 ng/ml within the first hours after CYPHER stent placement [33]. The concentration in the vascular wall is expected to be higher. The stimulation of TF expression by sirolimus (20 ng/ml) is also time-dependent (24 hours, 270% increase above control, Fig. 1B, n = 6, p < 0.01). Our result is in line with a recent observation by Guba *et al.*, showing that sirolimus stimulates TF expression in human endothelial cells [34].

**Figure 2:**
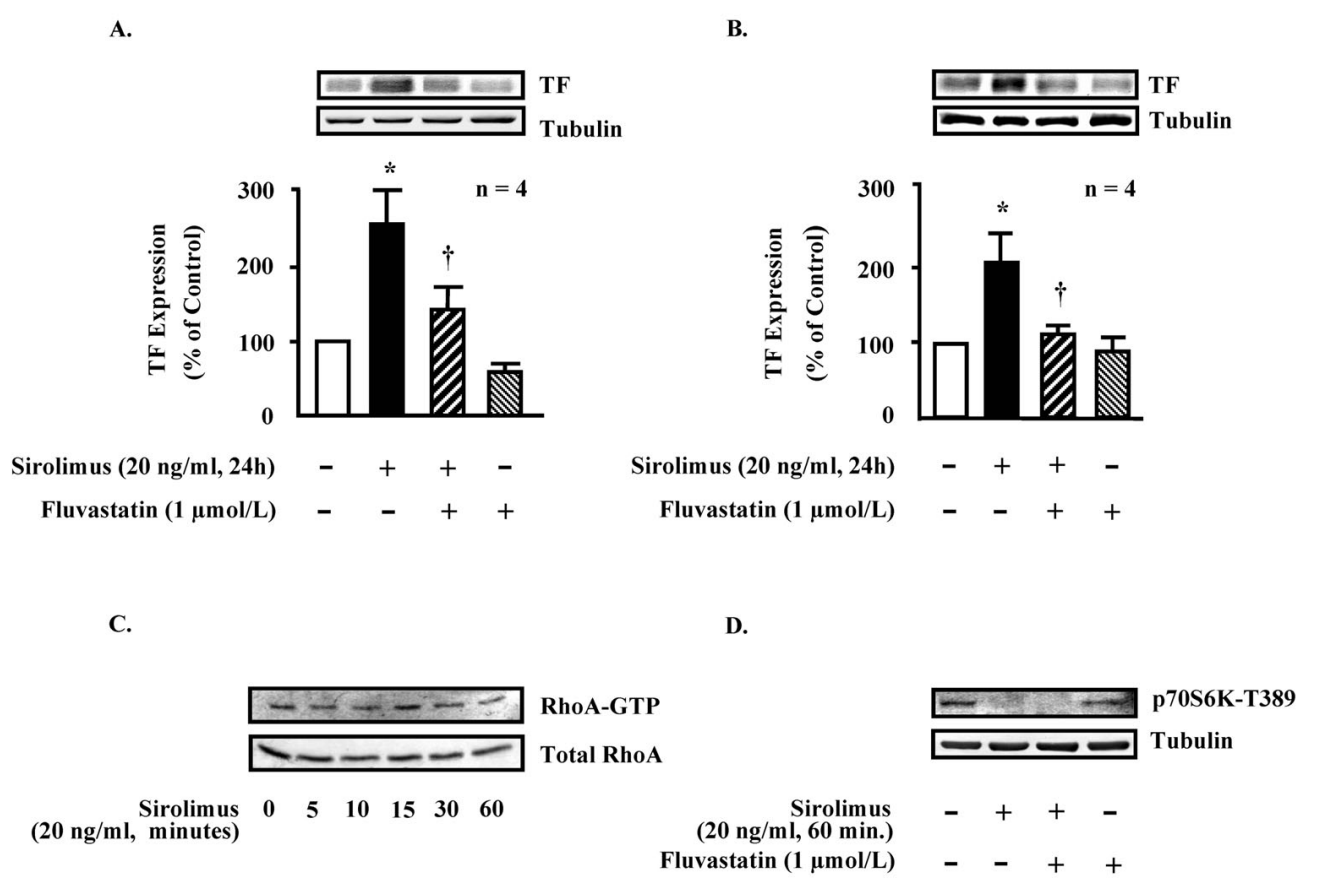
**Fluvastatin inhibits TF expression in SMC. **In human saphenous vein SMC (HSVSMC, panel A, n = 4) as well as in human aortic SMC (HAoSMC, panel B, n = 4) sirolimus (20 ng/ml, 24 hours) up-regulated TF expression which was significantly inhibited by fluvastatin (1 μmol/L). Basal activity of RhoA was not influenced by sirolimus (20 ng/ml, n = 3, panel C), while the basal activity of mTOR was fully inhibited by sirolimus (20 ng/ml, 1 hour, n = 3, panel D). * = p < 0.01 vs. control, ^† ^= p < 0.05 vs. sirolimus.

Furthermore, we showed that the induction of TF by sirolimus (20 ng/ml, 24 hours) was fully inhibited by the HMG-CoA reductase inhibitor fluvastatin (1 μmol/L) in human saphenous vein (Fig. 2A) and aorta SMCs (Fig. 2B) (n = 4, p < 0.05). Fluvastatin alone, however, did not significantly affect the basal TF expression in the cells (Fig. 2A and Fig. 2B). Statins exert many effects on vascular cells via inhibition of RhoA [20,35]. It is, however, unlikely that this mechanism explains the inhibitory effect of TF expression by fluvastatin. Firstly, a significant basal activity of RhoA was present in SMC (Fig. 2C), which was, however, not further stimulated by sirolimus (20 ng/ml) over 60 minutes (Fig. 2C). Secondly, fluvastatin alone did not inhibit basal TF expression (Fig. 2A and 2B), suggesting that basal TF expression is not mediated by RhoA. Results of our previous studies and others demonstrated that besides RhoA, p38mapk is also a positive regulator of TF expression in vascular endothelial cells and SMCs [15-17]. Our present study showed that no basal activity of p38mapk could be detected, and sirolimus (20 ng/ml, over 60 minutes) did not activate p38mapk in SMC. These results suggest that sirolimus enhances TF expression not through p38mapk and RhoA. Although the exact mechanism of statin-induced inhibition of TF expression by sirolimus is still obscure under this condition, our results support the clinical benefit of statins in patients with coronary stenting.

It is well described that sirolimus is a natural immunosuppressant which interferes with cellular functions via blockade of the protein kinase, mTOR [8] which further activates its downstream effector p70s6k by phosphorylating T389 residue [36]. In our present study, we showed a significant basal activity of mTOR in SMC as measured by p70s6k phosphorylation at T389 (Fig. 2D). The activity of mTOR was abolished by sirolimus (20 ng/ml, 1 hour treatment, Fig. 2D). Whether this data suggest an inhibitory effect of mTOR on TF expression needs further investigation. Further results demonstrate that fluvastatin does not reverse the inhibition of mTOR i.e. phosphorylation of p70s6k at T389 by sirolimus, nor it had any effect on basal mTOR activity in the cells (Fig. 2D), suggesting that statin inhibits TF expression not through regulation of mTOR.

**Figure 3:**
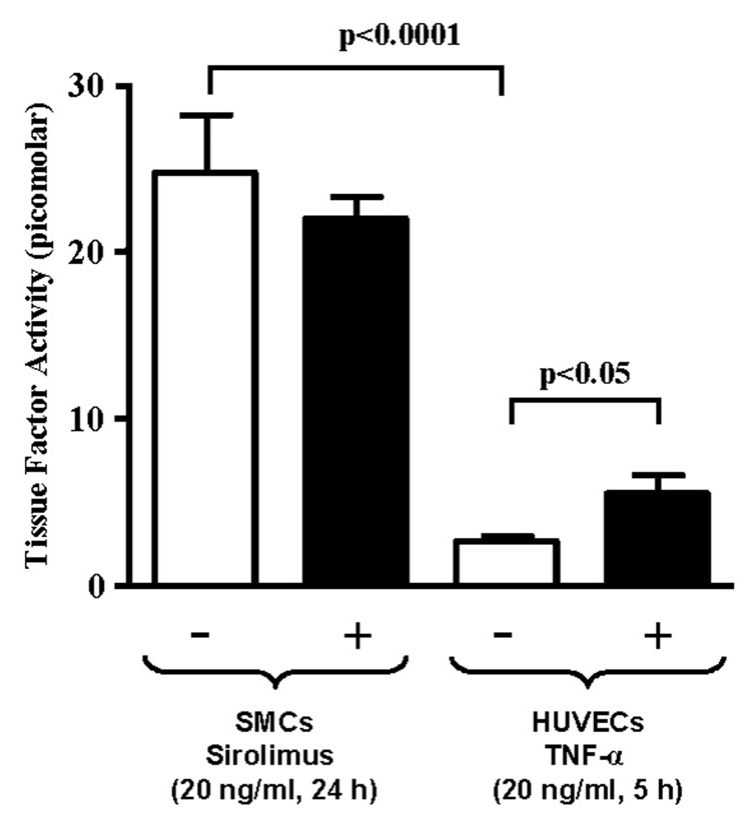
**Sirolimus does not stimulate TF release and activity from SMC. **Human SMC released much higher TF activity into the culture medium than endothelial cells (HUVECs) (p < 0.0001, n = 6–9). TF activity released from SMC was not further increased by sirolimus (20 ng/ml, 24 hours). The TF activity released from HUVECs was significantly stimulated by TNF-α (20 ng/ml; 5 hours, p < 0.05, n = 9).

Despite increased TF protein expression by sirolimus in SMC, the activity of TF released from SMC into the culture medium was not enhanced by sirolimus (Fig. 3). For the validity of the method, a control experiment using endothelial cells was performed. Endothelial cells had much lower TF activity than SMC (p < 0.0001; n = 6–9), that was significantly enhanced by TNF-α (20 ng/ml; 5 hours, p < 0.05, n = 9, Fig. 3)

## Conclusion

Taken together, our results demonstrate that although sirolimus stimulates TF expression in human SMC, it does not enhance TF activity released from the cells. The results support the safe profile of CYPHER stents observed by clinical trials. The inhibition of TF expression by fluvastatin favors clinical use of statins in patients undergoing coronary stenting.

## Competing interests

The author(s) declare that they have no competing interests.

## Authors' contributions

SZ and HV performed Western blot analysis for TF expression and activity and activation of mTOR. TG assisted us in human SMC culture and statistical analysis. XFM performed RhoA pull-down assay. ZY conceived and coordinated the study and drafted the manuscript. All authors read and approved the final manuscript.

## Pre-publication history

The pre-publication history for this paper can be accessed here:


